# Association between pan-immune-inflammation value and bone turnover markers in Chinese patients with osteoporotic fractures: a retrospective cross-sectional study

**DOI:** 10.3389/fmed.2025.1660376

**Published:** 2025-10-10

**Authors:** Jia-hao Wang, Meng-cheng Zhu, Chong Li, Guo-hua Wang, Ke Lu, Yan-ming Hao

**Affiliations:** ^1^Kunshan Biomedical Big Data Innovation Application Laboratory, Suzhou, Jiangsu, China; ^2^Department of Orthopedics, The First People’s Hospital of Kunshan, Gusu School, Nanjing Medical University, Suzhou, Jiangsu, China; ^3^Department of Orthopedics, Affiliated Kunshan Hospital of Jiangsu University, Suzhou, Jiangsu, China; ^4^Department of Orthopedics, Qiandeng First People’s Hospital of Kunshan, Suzhou, Jiangsu, China

**Keywords:** osteoporosis, fragility fracture, PIV, bone turnover markers, systemic inflammation, osteoimmunology

## Abstract

**Background:**

Systemic inflammation has been linked to impaired bone remodeling and may contribute to the risk of osteoporotic fractures (OPFs). This study examined the relationship between baseline pan-immune-inflammation value (PIV) and bone turnover markers (BTMs) in patients hospitalized for the surgical treatment of OPFs.

**Methods:**

In this retrospective cross-sectional study, 839 patients aged ≥50 years who were treated for osteoporotic fragility fractures between 2017 and 2024 were analyzed. PIV was calculated as (neutrophils × platelets × monocytes)/lymphocytes. BTMs included serum *β*-C-terminal telopeptide of type I collagen (β-CTX) and procollagen type I N-terminal propeptide (P1NP). Associations between log₂-transformed PIV and BTMs were assessed using multivariable generalized estimating equations (GEEs), adjusting for demographic, clinical, and biochemical factors. Smoothing spline models and threshold effect analyses were used to explore potential non-linear relationships. Subgroup analyses were conducted to examine effect modification.

**Results:**

The mean age of participants was 69.4 ± 10.9 years, with 70.9% being female. Mean *β*-CTX and P1NP levels were 0.54 ± 0.29 ng/mL and 58.1 ± 35.3 ng/mL, respectively, and the mean log₂PIV was 8.24 ± 1.28. Higher PIV levels were independently associated with lower BTMs. Specifically, each doubling of PIV was associated with a 4.46 ng/mL reduction in P1NP and a 0.05 ng/mL reduction in *β*-CTX (both *p* < 0.001). An inverted J-shaped association was observed, with the relationship plateauing at log₂PIV levels between approximately 8.3 and 10.3. The inverse association was more pronounced in individuals with hypertension or impaired renal function.

**Conclusion:**

Elevated PIV is independently and non-linearly associated with suppressed bone turnover, underscoring the role of systemic inflammation in the pathophysiology and management of osteoporosis.

## 1 Introduction

Osteoporosis is a systemic skeletal disorder characterized by reduced bone mass and deterioration of bone microarchitecture, leading to an increased risk of fractures (1). It affects 100 of millions worldwide, with incidence rates escalating due to an aging population and the associated healthcare burden (2, 3). Although hormonal and nutritional factors are well-recognized contributors, chronic inflammation has emerged as a key player in osteoporosis pathogenesis (4). Insights from osteoimmunology reveal that immune cells and pro-inflammatory cytokines such as tumor necrosis factor (TNF) and interleukin-6 (IL-6) can influence the activity of osteoclasts and osteoblasts (5, 6), particularly in postmenopausal women, where estrogen deficiency promotes pro-inflammatory responses (7). TNF-*α* and IL-6 disrupt skeletal remodeling by promoting RANKL-mediated bone resorption and suppressing osteoblast activity, thereby contributing to bone fragility in chronic inflammation (8). Elevated levels of inflammatory markers have been associated with decreased bone mineral density (BMD), increased fracture risk, and a greater overall burden of osteoporosis, especially in the context of chronic inflammatory conditions (9–12).

Inflammatory indices derived from routine blood counts have gained prominence as potential markers of osteoporosis risk. The pan-immune-inflammation value (PIV), calculated as (*neutrophils* × *platelets* × *monocytes*)/*lymphocytes*, captures the dynamic interplay between innate immune activation and adaptive immune suppression (13, 14). Initially developed in the context of oncology, elevated PIV has been linked to adverse outcomes in a range of inflammation-associated conditions (13, 14). Thus, considering the critical role of these immune cells in regulating bone metabolism (4, 5, 15), PIV may serve as a relevant marker of inflammation-related skeletal remodeling.

Simpler inflammation-based indices such as the neutrophil-to-lymphocyte ratio (NLR), platelet-to-lymphocyte ratio (PLR), and systemic immune-inflammation index (SII) have been linked to bone mineral density (BMD) and osteoporosis risk. Meta-analyses and cohort studies consistently show elevated levels of these markers in individuals with osteoporosis (16–18). Compared with these conventional markers (e.g., CRP, ESR, NLR, or PLR), PIV incorporates four distinct leukocyte subsets—neutrophils, monocytes, lymphocytes, and platelets—thereby offering a more integrated reflection of systemic immune-inflammatory activity. This comprehensive nature may capture the complexity of osteoimmune interactions more effectively and underscores the novelty of applying PIV in the context of bone remodeling (13, 19). In contrast, evidence on the association between PIV and bone health remains sparse and inconclusive. Two recent studies reported conflicting results regarding PIV levels in osteoporotic women, and neither studies investigated bone turnover markers (BTMs) (20, 21).

BTMs, such as *β*-C-terminal telopeptide of type I collagen (*β*-CTX) and procollagen type I N-terminal propeptide (P1NP), are widely utilized to assess skeletal remodeling activity, estimate fracture risk, and monitor therapeutic response (3, 22). β-CTX reflects bone resorption via type I collagen degradation by osteoclasts, while P1NP indicates bone formation through collagen synthesis by osteoblasts. Their simultaneous suppression suggests a low-turnover state with uncoupled remodeling, which weakens bone strength and elevates fracture risk (23). Chronic inflammation may suppress bone turnover through cytokine-mediated pathways, resulting in uncoupled remodeling and increased skeletal fragility (4, 5, 24, 25). We therefore hypothesized that elevated PIV is associated with lower BTM levels in patients with osteoporotic fractures, indicative of a low-turnover, inflammation-driven (“inflamm-aging”) osteoporosis phenotype. “Inflamm-aging” denotes chronic, low-grade inflammation associated with aging that disrupts bone homeostasis by enhancing catabolic signaling and suppressing anabolic activity, contributing to osteoporosis and other degenerative diseases (26).

To date, no study has directly examined the relationship between PIV and BTMs in patients with osteoporotic fractures. This study aimed to investigate this association in a Chinese cohort, assess potential nonlinear patterns and subgroup variations, and evaluate the clinical utility of PIV as a biomarker for inflammation-related changes in bone metabolism.

## 2 Materials and methods

### 2.1 Research participants and design

**Figure 1:**
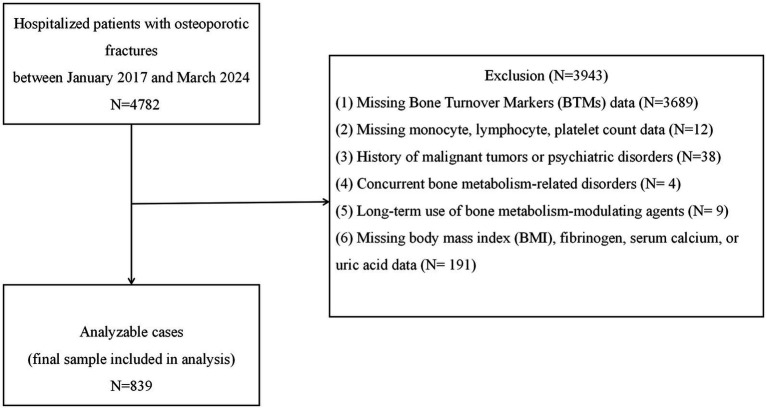
Flowchart of the study population selection process.

We conducted a retrospective cross-sectional analysis of electronic medical records from patients with osteoporotic fractures (OPFs) admitted to Kunshan Hospital, a tertiary Grade A facility in Jiangsu Province, between January 2017 and March 2024. The inclusion criteria were as follows: age ≥50 years; diagnosis of primary osteoporosis based on clinical or densitometric evidence; and the presence of an osteoporotic fragility fracture. Specifically, the study included patients with hip fractures (femoral neck, intertrochanteric, and subtrochanteric fractures), vertebral compression fractures (thoracic or lumbar), proximal humerus fractures, and distal radius (wrist) fractures, which represent the most common clinical types of osteoporotic fractures. Osteoporosis was defined as either (1) the occurrence of a low-trauma fracture with a bone mineral density (BMD) T-score ≤ − 2.5 at the spine or hip or (2) a BMD T-score of ≤ − 2.5 in the absence of secondary causes of bone loss, even without a documented fracture (20). The exclusion criteria included the presence of secondary bone metabolism disorders (e.g., hyperthyroidism, hyperparathyroidism, and chronic glucocorticoid use), malignancy, active rheumatic disease, severe psychiatric conditions, age <50 years, long-term osteoporosis treatment (e.g., bisphosphonates or parathyroid hormone analogs), or missing/outlier data for PIV or BTMs. Of the 4,782 fracture cases initially screened, 839 patients met all inclusion and exclusion criteria and were included in the final analysis. The patient selection process is outlined in Figure 1. As a hospital-based study in China, the findings offer region-specific insights, as differences in nutrition, inflammation, and healthcare access may affect osteoporosis risk and biomarker expression. This underscores the value of localized data for guiding diagnosis and treatment.

### 2.2 Exposure and outcome variables

Preoperative complete blood counts were obtained using the Sysmex XN-10 automated hematology analyzer (Sysmex Corp., Kobe, Japan), which provided neutrophil, lymphocyte, monocyte, and platelet counts (18, 27). The pan-immune-inflammation value (PIV) was calculated as (*neutrophils* × *platelets* × *monocytes*)/*lymphocytes*, following established methods (13, 14). Due to its right-skewed distribution, PIV was log₂-transformed (log₂PIV) prior to analysis and used as the primary exposure variable (20). Outcome variables were P1NP and *β*-CTX, assessed via electrochemiluminescence immunoassay (ECLIA) on a Roche Cobas 8,000 system. All tests were performed by certified technicians using standardized protocols.

### 2.3 Covariates

Covariates included age, sex, body mass index (BMI), smoking status, alcohol consumption, and the Charlson Comorbidity Index (CCI). Medical histories of hypertension and diabetes mellitus were also documented. Laboratory assessments encompassed serum calcium, uric acid (UA), blood urea nitrogen (BUN), creatinine (Cr), and liver enzymes alanine aminotransferase (ALT) and aspartate aminotransferase (AST). ALT and AST levels were measured using enzymatic colorimetry; BUN and Cr were determined via enzymatic methods using the Beckman AU5800 analyzer; and UA was assessed using the uricase–peroxidase method. BMI was calculated as weight in kilograms divided by height in meters squared (kg/m^2^). Smoking was defined as current or former use within the past 12 months, while alcohol consumption was defined as drinking at least once per week in the past year. All laboratory tests were conducted on fasting venous blood samples collected within 8 h prior to surgery and processed by certified laboratory personnel according to standard operating procedures (SOPs).

### 2.4 Statistical analyses

Continuous variables with approximately normal distributions were summarized as means ± standard deviation (*SD*), while skewed continuous variables were reported as medians with interquartile ranges (Q1 and Q3). Categorical variables were expressed as counts and percentages. For between-group comparisons, Student’s *t*-test was used for normally distributed continuous variables, the Mann–Whitney U-test for non-normally distributed variables and Pearson’s chi-square test or Fisher’s exact test for categorical variables, as appropriate. To examine trends across varying levels of systemic inflammation, baseline characteristics were stratified by quartiles of log₂-transformed PIV (log₂PIV). Univariate comparisons across these quartiles were conducted using a one-way ANOVA for normally distributed variables and the Kruskal–Wallis test for non-normally distributed variables.

Univariate linear regression analyses were initially conducted to examine the associations between each bone turnover marker (*β*-CTX and P1NP, as dependent variables) and individual covariates, including age, sex, BMI, lifestyle factors, comorbidities, serum calcium, UA, BUN, Cr, ALT, AST, and others. Covariates with a *p*-value of ≤0.10 in the univariate analysis, or those that altered the estimated association between PIV and BTMs by ≥10%, were considered for inclusion in the multivariable regression models (24). Multicollinearity among covariates was assessed using variance inflation factors (VIFs), with a VIF of <5 considered acceptable, and no significant collinearity was observed, including for liver enzymes (ALT and AST) and other laboratory parameters.

Generalized estimating equations (GEEs) with an identity link function were used to evaluate the independent associations between log₂-transformed PIV (log₂PIV) and each bone turnover marker. Three sequential models were developed: Model 1 assessed the unadjusted (univariate) association; Model 2 was adjusted for key demographic and clinical variables, including age, sex, BMI, smoking status, alcohol use, Charlson Comorbidity Index (CCI), hypertension, and diabetes; Model 3 was fully adjusted, incorporating laboratory parameters, serum calcium, UA, BUN, Cr, ALT, and AST into Model 2. These additional covariates in Model 3 accounted for nutritional and metabolic factors potentially affecting both systemic inflammation and bone turnover. Results are presented as beta coefficients (*β*) with 95% confidence intervals (CIs) and a *p*-values for the association between log₂PIV and BTM levels. A negative *β* indicates lower BTMs with higher PIV.

To investigate potential non-linear relationships between PIV and BTMs, we utilized generalized additive models (GAMs) and smoothing spline plots (27). If a non-linear pattern was suggested, we performed a two-piece linear regression (threshold effect analysis) to identify a potential inflection point (knot) in the PIV-BTM relationship (24). Separate linear regressions were then fitted on either side of the identified inflection point, and the slopes were compared. A log-likelihood ratio test was used to assess whether the two-piece model provided a significantly better fit than a single linear model, indicating the presence of a threshold effect. To ensure the robustness of the identified cut-point, the threshold was further validated using bootstrap resampling with 1,000 iterations.

Stratified analyses were performed to examine whether the association between PIV and BTMs was consistent across clinically relevant subgroups. Factors were selected from baseline covariates based on biological plausibility and prior literature, including demographics (age, sex, and BMI), lifestyle (smoking and drinking), comorbidities (hypertension, diabetes, and CCI), and biochemical parameters (Ca, UA, UN, Cr, ALT, and AST). Age was stratified as ≤70 vs. >70 years; BMI as <25, 25–29.9, and ≥30 kg/m^2^; and laboratory parameters using clinically relevant cutoff values (e.g., AST < 40 vs. ≥40 U/L, UA < 420 vs. ≥420 μmol/L, Ca < 2.3 vs. ≥2.3 mmol/L). Associations were re-estimated within each subgroup using the fully adjusted Model 3, and interaction terms were tested in GEE models, with *p* for interaction <0.05 indicating significant effect modification.

All statistical analyses were conducted using R software (version 4.0.5; R Foundation for Statistical Computing, Vienna, Austria) and EmpowerStats (X and Y Solutions, Boston, MA). A two-tailed *p*-value of <0.05 was considered statistically significant. Due to the exploratory nature of the subgroup analyses, interaction effects were interpreted with caution.

## 3 Results

### 3.1 Participants’ baseline characteristics

A total of 839 patients with osteoporotic fractures were included in the analysis (mean age: 69.42 ± 10.92 years; 70.9% female). The mean log₂PIV was 8.24 ± 1.28. Baseline characteristics stratified by PIV quartiles (Q1–Q4) are summarized in Table 1. Significant differences in several inflammation-related laboratory parameters were observed across quartiles. Serum UA levels increased steadily with higher PIV, from 262.3 ± 76.9 μmol/L in Q1 to 302.9 ± 91.5 μmol/L in Q4 (*p* < 0.001). ALT and AST levels were also elevated in Q4 compared to lower quartiles (both *p* < 0.001), though still within normal limits. Notably, bone turnover markers declined with increasing PIV. Mean P1NP decreased from 68.06 ± 33.69 ng/mL in Q1 to 51.84 ± 44.84 ng/mL in Q4 (*p* < 0.001), and *β*-CTX dropped from 0.64 ± 0.31 ng/mL to 0.45 ± 0.28 ng/mL (*p* < 0.001). These trends suggest that higher systemic inflammation, as indicated by elevated PIV, is associated with suppressed bone formation and resorption.

**Table 1:** Characteristics of study participants by PIV quartile.

Characteristics	Mean ± *SD*/*N* (%)					*P*-value
	Total (*n* = 839)	Q1 (*n* = 210)	Q2 (*n* = 209)	Q3 (*n* = 210)	Q4 (*n* = 210)	
Log2PIV	8.24 ± 1.28	6.64 ± 0.58	7.83 ± 0.23	8.60 ± 0.23	9.88 ± 0.73	<0.001
Age, years	69.42 ± 10.92	70.06 ± 11.16	68.48 ± 11.03	69.20 ± 10.78	69.92 ± 10.72	0.43
UA, μmol/L	281.73 ± 89.76	262.28 ± 76.92	280.44 ± 91.62	281.24 ± 93.98	302.94 ± 91.48	<0.001
UN, mmol/L	6.23 ± 5.09	6.58 ± 9.44	5.96 ± 2.12	6.14 ± 2.10	6.26 ± 2.39	0.47
Cr, μmol/L	63.26 ± 24.50	61.48 ± 21.36	62.60 ± 19.64	63.96 ± 24.01	64.98 ± 31.36	0.40
ALT, U/L	22.60 ± 16.05	19.16 ± 10.88	23.28 ± 14.96	21.50 ± 11.84	26.45 ± 22.88	<0.001
AST, U/L	25.72 ± 15.58	22.67 ± 11.17	24.15 ± 9.32	24.40 ± 9.19	31.65 ± 25.09	<0.001
Ca, mmol/L	2.22 ± 0.12	2.22 ± 0.11	2.23 ± 0.12	2.23 ± 0.13	2.22 ± 0.12	0.69
P1NP, ng/mL	58.06 ± 35.25	68.06 ± 33.69	57.00 ± 27.08	55.35 ± 30.98	51.84 ± 44.84	<0.001
β-CTX, ng/mL	0.54 ± 0.29	0.64 ± 0.31	0.55 ± 0.27	0.52 ± 0.26	0.45 ± 0.28	<0.001
Sex, *N* (%)						0.45
Female	595 (70.92%)	157 (74.76%)	142 (67.94%)	150 (71.43%)	146 (69.52%)	
Male	244 (29.08%)	53 (25.24%)	67 (32.06%)	60 (28.57%)	64 (30.48%)	
CCI, *N* (%)						0.05
0	739 (88.08%)	191 (90.95%)	177 (84.69%)	178 (84.76%)	193 (91.90%)	
1	79 (9.42%)	16 (7.62%)	25 (11.96%)	22 (10.48%)	16 (7.62%)	
≥2	21 (2.50%)	3 (1.43%)	7 (3.35%)	10 (4.76%)	1 (0.48%)	
BMI, *N* (%)						0.16
<25	594 (70.80%)	141 (67.14%)	147 (70.33%)	155 (73.81%)	151 (71.90%)	
≥25, <30	219 (26.10%)	58 (27.62%)	53 (25.36%)	53 (25.24%)	55 (26.19%)	
≥30	26 (3.10%)	11 (5.24%)	9 (4.31%)	2 (0.95%)	4 (1.90%)	
Smoke, *N* (%)						0.18
No	803 (95.71%)	206 (98.10%)	196 (93.78%)	200 (95.24%)	201 (95.71%)	
Yes	36 (4.29%)	4 (1.90%)	13 (6.22%)	10 (4.76%)	9 (4.29%)	
Drink, *N* (%)						0.20
No	813 (96.90%)	208 (99.05%)	202 (96.65%)	201 (95.71%)	202 (96.19%)	
Yes	26 (3.10%)	2 (0.95%)	7 (3.35%)	9 (4.29%)	8 (3.81%)	
Diabetes, *N* (%)						0.16
No	802 (95.59%)	201 (95.71%)	199 (95.22%)	196 (93.33%)	206 (98.10%)	
Yes	37 (4.41%)	9 (4.29%)	10 (4.78%)	14 (6.67%)	4 (1.90%)	
Hypertension, *N* (%)						0.73
No	717 (85.46%)	177 (84.29%)	176 (84.21%)	180 (85.71%)	184 (87.62%)	
Yes	122 (14.54%)	33 (15.71%)	33 (15.79%)	30 (14.29%)	26 (12.38%)	

PIV, pan-immune-inflammation value; SD, standard deviation; Q1, first quartile; Q2, second quartile; Q3, third quartile; Q4, fourth quartile; UA, uric acid; UN, urea nitrogen; Cr, creatinine; ALT, alanine aminotransferase; AST, aspartate aminotransferase; Ca, calcium; P1NP, procollagen type I N-terminal propeptide; β-CTX, β-C-terminal telopeptide of type I collagen; CCI, Charlson Comorbidity Index; BMI, body mass index.

### 3.2 Univariate analysis of factors associated with BTMs

In the univariate analysis (Table 2), several variables showed associations with bone turnover markers. Higher ALT and AST levels were modestly linked to lower P1NP concentrations (ALT: *β* = −0.00, 95% *CI*: −0.00 to −0.00, *p* = 0.003; AST: *β* = −0.00, *p* = 0.001 per 1 U/L increase). Increased UA was significantly associated with reduced P1NP (*β* = −0.00 ng/mL per μmol/L, *p* = 0.01) and showed a non-significant trend toward lower β-CTX (*p* = 0.13). In contrast, higher Cr was positively associated with both P1NP (*p* < 0.001) and β-CTX (*p* = 0.002). No other covariates were significantly associated with bone turnover markers in the univariate analysis.

**Table 2:** Univariate regression analysis for factors associated with P1NP and *β*-CTX.

Characteristics	Statistics	P1NP	β-CTX
		*β* (95% *CI*) *P*-value	
Age, years	69.42 ± 10.92	−0.00 (−0.00, 0.00) 0.69	0.06 (−0.16, 0.28) 0.58
ALT, U/L	22.60 ± 16.05	−0.00 (−0.00, −0.00) 0.003	−0.06 (−0.21, 0.09) 0.42
AST, U/L	25.72 ± 15.58	−0.00 (−0.00, −0.00) 0.001	0.02 (−0.13, 0.17) 0.80
UN, mmol/L	6.23 ± 5.09	0.00 (−0.00, 0.01) 0.09	0.50 (0.04, 0.97) 0.03
UA, μmol/L	281.73 ± 89.76	−0.00 (−0.00, −0.00) 0.01	−0.02 (−0.05, 0.01) 0.13
Cr, μmol/L	63.26 ± 24.50	0.00 (0.00, 0.00) 0.0001	0.15 (0.06, 0.25) 0.002
Ca, mmol/L	2.22 ± 0.12	0.11 (−0.05, 0.27) 0.17	12.34 (−7.21, 31.89) 0.21
Sex, *N* (%)			
Female	595 (70.92%)	Reference	Reference
Male	244 (29.08%)	0.03 (−0.01, 0.08) 0.14	1.83 (−3.43, 7.08) 0.50
CCI, *N* (%)			
0	739 (88.08%)	Reference	Reference
1	79 (9.42%)	−0.00 (−0.07, 0.07) 0.99	3.61 (−4.58, 11.79) 0.39
≥2	21 (2.50%)	−0.02 (−0.14, 0.11) 0.77	5.26 (−10.03, 20.56) 0.50
BMI, *N* (%)			
<25	594 (70.80%)	Reference	Reference
> = 25, <30	219 (26.10%)	−0.04 (−0.09, 0.00) 0.07	−0.54 (−6.00, 4.93) 0.85
> = 30	26 (3.10%)	0.04 (−0.08, 0.15) 0.52	3.20 (−10.66, 17.06) 0.65
Smoke, *N* (%)			
No	803 (95.71%)	Reference	Reference
Yes	36 (4.29%)	−0.00 (−0.10, 0.09) 0.92	−0.72 (−12.50, 11.06) 0.90
Drink, *N* (%)			
No	813 (96.90%)	Reference	Reference
Yes	26 (3.10%)	−0.04 (−0.15, 0.07) 0.47	−1.55 (−15.32, 12.22) 0.83
Diabetes, *N* (%)			
No	802 (95.59%)	Reference	Reference
Yes	37 (4.41%)	0.03 (−0.07, 0.12) 0.59	5.51 (−6.11, 17.13) 0.35
Hypertension, *N* (%)			
No	717 (85.46%)	Reference	Reference
Yes	122 (14.54%)	0.00 (−0.05, 0.06) 0.87	1.57 (−5.20, 8.34) 0.65

CI, confidence interval; UA, uric acid; UN, urea nitrogen; Cr, creatinine; ALT, alanine aminotransferase; AST, aspartate aminotransferase; Ca, calcium; P1NP, procollagen type I N-terminal propeptide; β-CTX, β-C-terminal telopeptide of type I collagen; CCI, Charlson comorbidity index; BMI, body mass index.

### 3.3 Association between PIV and bone turnover markers

Multivariable regression models confirmed a significant independent association between higher PIV and lower levels of both bone formation and resorption markers (Table 3). In the unadjusted model (Model 1), log₂PIV was strongly and inversely associated with *β*-CTX (*β* = −0.05 ng/mL per 1-unit increase, 95% *CI*: −0.07 to −0.04, *p* < 0.001) and P1NP (*β* = −4.41 ng/mL, 95% *CI*: −6.26 to −2.57, *p* < 0.001). Each doubling of PIV (i.e., a 1-unit increase in log₂PIV) corresponded to an approximate reduction of 0.05 ng/mL in *β*-CTX and 4.4 ng/mL in P1NP. These associations remained consistent after adjusting for age, sex, BMI, smoking, alcohol use, and comorbidities in Model 2 and persisted after further adjusting for calcium, UA, renal function, and liver enzymes in Model 3. In the fully adjusted model, log₂PIV continued to show significant inverse associations with both *β*-CTX (*β* = −0.05, 95% *CI*: −0.06 to −0.03) and P1NP (*β* = −4.46, 95% *CI*: −6.36 to −2.56), with both *p* < 0.001. These results highlight a strong and independent inverse relationship between systemic inflammation and bone turnover activity.

**Table 3:** Multivariable linear regression analyses of log₂PIV associated with β-CTX and P1NP levels.

*β* (95% *CI*) *P*-value	Model 1^a^ *β* (95% *CI*) *P*-value	Model 2^b^ *β* (95% *CI*) *P*-value	Model 3^c^ *β* (95% *CI*) *p*-value
β-CTX	−0.05 (−0.07, −0.04) < 0.001	−0.05 (−0.07, −0.04) < 0.001	−0.05 (−0.06, −0.03) < 0.001
P1NP	−4.41 (−6.26, −2.57) < 0.001	−4.43 (−6.29, −2.57) < 0.001	−4.46 (−6.36, −2.56) < 0.001

a No adjustment.

b Adjusted for age, sex, BMI, smoke, drink, CCI, diabetes, and hypertension.

c Adjusted for age, sex, BMI, smoke, drink, CCI, diabetes, hypertension, Ca, UA, UN; Cr, ALT, and AST.

PIV, pan-immune-inflammation value; CI, confidence interval; UA, uric acid; UN, urea nitrogen; Cr, creatinine; ALT, alanine aminotransferase; AST, aspartate aminotransferase; Ca, calcium; P1NP, procollagen type I N-terminal propeptide; β-CTX, β-C-terminal telopeptide of type I collagen; CCI, Charlson comorbidity index; BMI, body mass index.

### 3.4 Spline smoothing plot and threshold analysis

**Figure 2:**
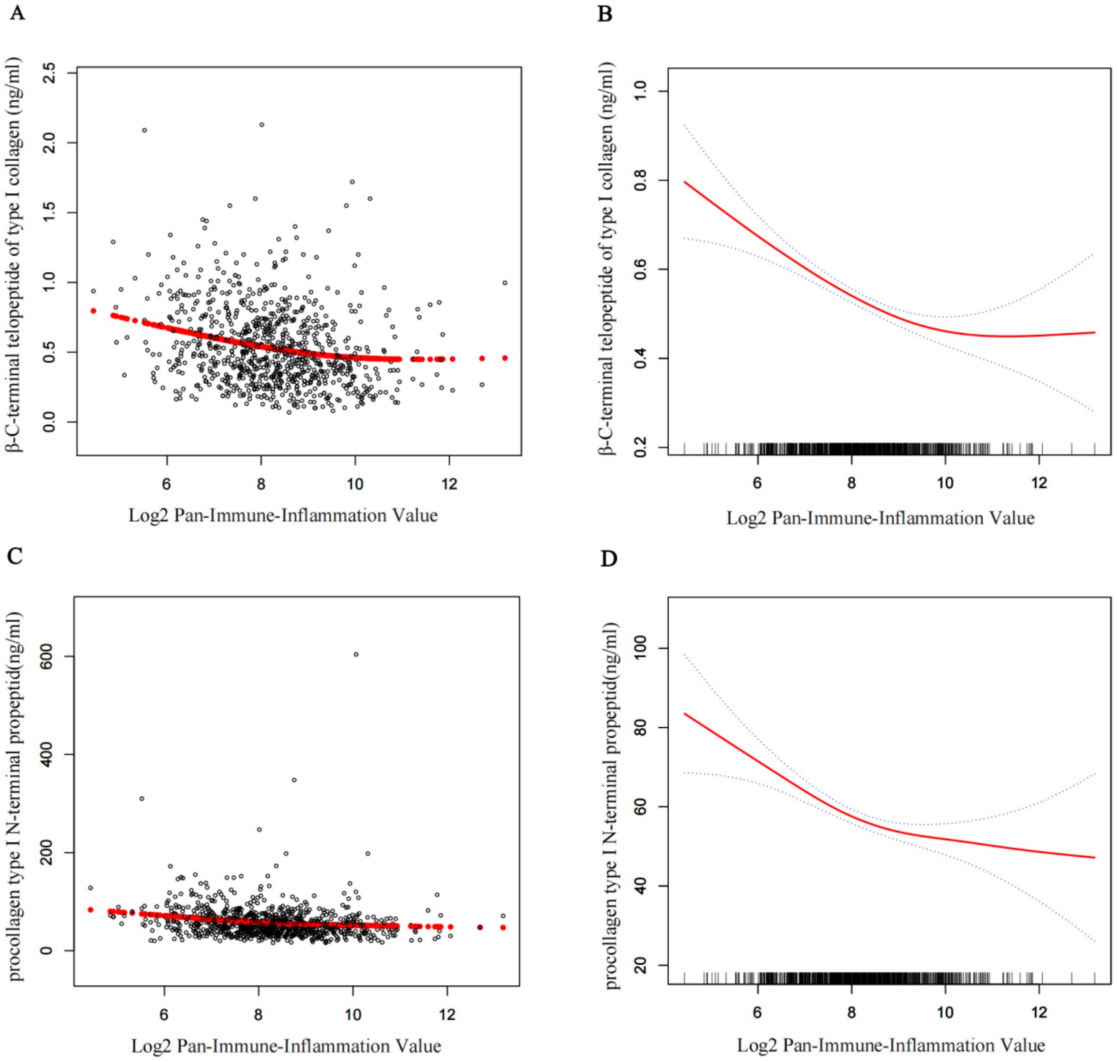
Smoothed curves showing the association between log2PIV and BTMs. **(A,C)** Each black point represents a single participant sample. **(B,D)** Solid red line represents the smooth curve fit between variables. Blue bands represent the 95% confidence interval from the fit. Age, sex, BMI, smoke, drink, CCI, diabetes, hypertension, Ca, UA, UN, Cr, ALT, and AST were adjusted. PIV, pan-immune-inflammation value; BTMs, bone turnover makers; UA, uric acid; UN, urea nitrogen; Cr, creatinine; ALT, alanine aminotransferase; AST, aspartate aminotransferase; Ca, calcium; P1NP, procollagen type I N-terminal propeptide; *β*-CTX, β-C-terminal telopeptide of type I collagen; CCI, Charlson Comorbidity Index; BMI, body mass index.

Figure 2 displays the fully adjusted smooth curves depicting the relationship between log₂PIV and bone turnover markers using GAMs. Both *β*-CTX (Figures 2A,B) and P1NP (Figures 2C,D) showed significant non-linear, inverted J-shaped associations. The curves inflected at log₂PIV values of approximately 10.31 for *β*-CTX and 8.26 for P1NP (Table 4), beyond which the associations plateaued. Below these thresholds, a 1-unit increase in log₂PIV was significantly associated with lower marker levels (β = −0.06 ng/mL for β-CTX, *p* < 0.001; β = −8.50 ng/mL for P1NP, *p* < 0.001), whereas above them the associations were no longer significant. The differences in slope before and after the threshold were statistically significant for both markers (*p* = 0.01), confirming the presence of threshold effects. Nearly 15% of patients had log₂PIV > 10.3, within which β-CTX values remained consistently low. In contrast, the threshold for P1NP (log₂PIV = 8.3, equivalent to raw PIV = 325) was close to the cohort median, suggesting that even moderate levels of systemic inflammation are linked to reduced bone formation. Bootstrap resampling confirmed the stability of the identified inflection points.

**Table 4:** Threshold analyses examining the relationship between log2PIV and BTMs.

*β* (95% *CI*) *P*-value	Model 3^a^	
	β-CTX *β* (95% *CI*) *P*-value	P1NP *β* (95% *CI*) *P*-value
Model A^b^		
One line slope	−0.05 (−0.06, −0.03) < 0.001	−4.46 (−6.36, −2.56) < 0.001
Model B^c^		
log2PIV turning point (K)	10.31	8.26
<K	−0.06 (−0.08, −0.04) < 0.001	−8.50 (−12.14, −4.85) < 0.001
>K	0.08 (−0.01, 0.17) 0.09	−0.60 (−4.12, 2.93) 0.74
Difference in slopes (Slope₂ – Slope₁)	7.90 (1.81, 13.99) 0.01	0.14 (0.04, 0.23) 0.01
LRT^d^	0.005	0.01

a Adjusted for age, sex, BMI, smoke, drink, CCI, diabetes, hypertension, Ca, UA, UN; Cr, ALT, and AST.

b Linear analysis, a *p* < 0.05 indicates a linear relationship.

c Non-linear analysis.

d *p* < 0.05 means Model B is significantly different from Model A, which indicates a non-linear relationship.

PIV, pan-immune-inflammation value; BTMs, bone turnover markers; UA, uric acid; CI, confidence interval; UN, urea nitrogen; Cr, creatinine; ALT, alanine aminotransferase; AST, aspartate aminotransferase; Ca, calcium; P1NP, procollagen type I N-terminal propeptide; β-CTX, β-C-terminal telopeptide of type I collagen; CCI, Charlson comorbidity index; BMI, body mass index; LRT, likelihood ratio test.

### 3.5 Subgroup analysis

We examined whether the inverse association between log₂PIV and bone turnover markers was consistent across patient subgroups (Table 5). Overall, the relationship remained robust across most subgroups. For example, in patients aged ≤70 years, higher PIV was significantly associated with lower *β*-CTX and P1NP levels (both *p* < 0.001), and similar associations were observed in those >70 years. No significant interactions were detected for age, sex, BMI, smoking, or diabetes (all *P* for interaction >0.1), indicating a generally consistent pattern. Nevertheless, hypertension significantly modified the association (*P* for interaction = 0.03). Among hypertensive patients, the inverse relationship was stronger (*β* = −0.10 for β-CTX; *β* = −7.63 for P1NP, both *p* < 0.001), potentially reflecting greater underlying vascular inflammation. Renal function also influenced the association. In individuals with normal UN (<7.5 mmol/L), PIV was significantly associated with lower marker levels (*β* = −0.06 for β-CTX; *β* = −5.77 for P1NP, both *p* < 0.001), whereas no significant relationship was observed in those with elevated UN (*P* for interaction = 0.007). Similarly, the association remained significant in patients with Cr < 115 μmol/L but was absent in those with Cr ≥ 115 μmol/L (*P* for interaction = 0.01), potentially due to impaired marker metabolism in kidney dysfunction. These findings suggest that, while the inverse relationship between PIV and bone turnover is generally stable, it may be more pronounced in patients with hypertension and weakened in those with impaired renal function.

**Table 5:** Subgroup analyses examine the relationship between log2PIV and BTMs.

Subgroup	*N*	β-CTX *β* (95% *CI*) *P*-value	*P* for interaction	P1NP *β* (95% *CI*) *P*-value	*P* for interaction
Age, years			0.62		0.22
>50, ≤70	460	−0.06 (−0.08, −0.04) < 0.001		−5.49 (−7.74, −3.25) < 0.001	
>70	379	−0.05 (−0.07, −0.03) < 0.001		−3.18 (−6.21, −0.15) 0.04	
Sex, *N*			0.84		0.22
Female	595	−0.05 (−0.07, −0.04) < 0.001		−5.12 (−7.02, −3.23) < 0.001	
Male	244	−0.05 (−0.08, −0.02) 0.002		−2.49 (−6.96, 1.99) 0.28	
BMI, kg/m2			0.98		0.87
<25	594	−0.05 (−0.07, −0.03) < 0.001		−4.09 (−6.40, −1.78) < 0.001	
≥25, <30	219	−0.06 (−0.08, −0.03) < 0.001		−5.13 (−8.38, −1.88) 0.002	
≥30	26	−0.05 (−0.14, 0.03) 0.22		−5.40 (−12.91, 2.12) 0.17	
Smoke, *N*			0.87		0.54
No	803	−0.05 (−0.07, −0.04) < 0.001		−4.30 (−6.20, −2.40) < 0.001	
Yes	36	−0.06 (−0.14, 0.03) 0.18		−7.35 (−14.47, −0.23) 0.05	
Drink, *N*			0.34		0.88
No	813	−0.05 (−0.07, −0.04) < 0.001		−4.43 (−6.31, −2.56) < 0.001	
Yes	26	−0.00 (−0.13, 0.12) 0.96		−3.46 (−14.67, 7.75) 0.55	
CCI, *N*			0.96		0.26
0	739	−0.05 (−0.07, −0.04) < 0.001		−4.07 (−6.03, −2.11) < 0.001	
1	79	−0.05 (−0.09, 0.00) 0.06		−9.39 (−14.80, −3.98) 0.001	
≥2	21	−0.07 (−0.18, 0.04) 0.26		2.08 (−16.62, 20.78) 0.83	
Hypertension, *N*			**0.03**		0.20
No	717	−0.05 (−0.06, −0.03) < 0.001		−3.96 (−5.99, −1.94) < 0.001	
Yes	122	−0.10 (−0.13, −0.06) < 0.001		−7.63 (−12.02, −3.24) < 0.001	
Diabetes, *N*			0.73		0.16
No	802	−0.05 (−0.07, −0.04) < 0.001		−4.18 (−6.06, −2.30) < 0.001	
Yes	37	−0.07 (−0.15, 0.02) 0.12		−12.48 (−22.02, −2.95) 0.015	
AST, U/L			0.88		0.69
<40	766	−0.05 (−0.07, −0.04) < 0.001		−4.30 (−6.34, −2.25) < 0.001	
≥40	73	−0.05 (−0.08, −0.01) 0.01		−5.41 (−9.32, −1.49) 0.01	
ALT, U/L			0.86		0.69
<40	771	−0.05 (−0.07, −0.04) < 0.001		−4.29 (−6.27, −2.30) < 0.001	
≥40	68	−0.06 (−0.09, −0.02) 0.003		−5.54 (−9.82, −1.26) 0.01	
UN, mmol/L			**0.007**		**0.005**
<7.5	680	−0.06 (−0.08, −0.05) < 0.001		−5.77 (−7.48, −4.06) < 0.001	
≥7.5	159	−0.01 (−0.05, 0.03) 0.53		0.87 (−5.41, 7.16) 0.79	
UA, μmol/L			0.36		0.58
<420	780	−0.05 (−0.07, −0.04) < 0.001		−4.53 (−6.42, −2.64) < 0.001	
≥420	59	−0.03 (−0.11, 0.06) 0.54		−2.33 (−10.82, 6.16) 0.59	
Cr, μmol/L			0.16		**0.01**
<115	819	−0.05 (−0.06, −0.04) < 0.001		−3.90 (−5.71, −2.08) < 0.001	
≥115	20	−0.11 (−0.25, 0.02) 0.12		−17.51 (−36.04, 1.01) 0.08	
Ca, mmol/L			0.08		0.29
<2.3	597	−0.04 (−0.06, −0.03) < 0.001		−3.78 (−6.13, −1.44) 0.002	
≥2.3, <2.8	242	−0.07 (−0.10, −0.05) < 0.001		−5.98 (−8.73, −3.22) < 0.001	

Adjusted for age, sex, BMI, smoke, drink, CCI, diabetes, hypertension, Ca, UA, UN; Cr, ALT, and AST. PIV, pan-immune-inflammation value; BTMs, bone turnover makers; UA, uric acid; CI, confidence interval; UN, urea nitrogen; Cr, creatinine; ALT, alanine aminotransferase; AST, aspartate aminotransferase; Ca, calcium; P1NP, procollagen type I N-terminal propeptide; β-CTX, β-C-terminal telopeptide of type I collagen; CCI, Charlson Comorbidity Index; BMI, body mass index. Bold values indicate *p* < 0.05 for interaction terms.

## 4 Discussion

In this study of 839 patients with osteoporotic fractures, we found that elevated PIV, a composite index derived from peripheral blood counts, was independently associated with lower serum levels of bone turnover markers (*β*-CTX and P1NP), even after adjusting for demographic, clinical, and biochemical variables. To our knowledge, this is the first study to demonstrate a direct link between PIV and bone remodeling activity in the context of osteoporosis. These results support the hypothesis that chronic systemic inflammation, as indicated by higher PIV, may suppress bone turnover, aligning with the concept of “inflamm-aging” in skeletal health (5, 28). This suppressed remodeling state—characterized by reduced bone formation and resorption—may contribute to skeletal fragility and fracture risk (4, 22). Previous studies using simpler indices such as NLR, PLR, and SII have similarly suggested a relationship between inflammation and impaired bone turnover.

Our findings align with previous research on the relationship between inflammation and bone metabolism. For example, Zhou et al. reported that the systemic inflammatory response index (SIRI) was inversely associated with both *β*-CTX and P1NP in Chinese patients with osteoporotic fractures, showing effect sizes similar to those observed in our study (24). Similarly, Xu et al. found negative correlations between NLR and MLR with BTMs, while PLR showed a modest positive relationship (27). This finding may be attributed to the fact that platelets release anabolic factors such as PDGF and TGF-β (15, 29), potentially explaining the differing effect of PLR. Since PIV incorporates neutrophils, monocytes, lymphocytes, and platelets, the pro-resorptive effects of neutrophils and monocytes, along with reduced lymphocyte counts, appear to outweigh any anabolic contribution from platelets, resulting in an overall inverse relationship between PIV and bone turnover. This is biologically plausible, as neutrophils and monocytes produce cytokines such as IL-1, IL-6, and TNF-*α*, which stimulate osteoclastogenesis (4, 30), while lymphopenia may reflect diminished osteoprotective T-cell activity (5, 31, 32). These myeloid cells also contribute to oxidative stress and matrix degradation, while lymphopenia may disrupt regulatory T-cell networks essential for osteoblast support, reinforcing a catabolic inflammatory state (33).

Notably, even high platelet counts did not offset the suppression of bone turnover observed at elevated PIV levels, as confirmed in our threshold effect analysis. Interestingly, we identified a non-linear relationship between PIV and bone turnover markers. Beyond a log₂PIV range of approximately 8.3–10.3 (equivalent to PIV values of 300–1,200), BTM levels plateaued at low values despite further increases in inflammation. This may help explain the findings of Demir et al. (21), who found no significant difference in PIV between osteoporotic and control women; if most participants had PIV levels beyond the threshold, bone turnover may have already been maximally suppressed. The threshold for *β*-CTX (log₂PIV = 10.3) aligns with the upper 10% of PIV values observed in general populations (14), indicating a potential “inflammatory saturation point” beyond which osteoclast activity becomes markedly inhibited. In contrast, the lower threshold for P1NP (log₂PIV = 8.3) suggests that bone formation is more sensitive to inflammation, showing earlier suppression than resorption. This pattern supports a model in which early inflammation leads to uncoupled bone resorption (34), while chronic inflammation causes global suppression (4, 22). Comparable turnover dynamics are seen in chronic inflammatory diseases such as rheumatoid arthritis, where advanced stages are characterized by global suppression of BTMs, even in the presence of localized bone erosions (12, 35).

Clinically, PIV may serve as a biomarker for identifying an “inflamed” osteoporotic phenotype—patients with impaired bone quality and higher fracture risk despite similar BMD. These individuals may respond poorly to anabolic treatments unless inflammation is addressed (6, 22, 30). Elevated neutrophils or monocytes with lymphopenia at admission may indicate high PIV and suppressed bone turnover, suggesting the need to consider anti-inflammatory or immunomodulatory approaches. Importantly, therapies targeting pro-inflammatory cytokines, such as TNF or IL-6 inhibitors, have demonstrated the potential for reducing inflammation-related bone loss (34, 36). Although observational, our findings raise a therapeutic possibility: lowering PIV via infection control, comorbidity management, or anti-inflammatory therapies may help restore bone turnover. These hypotheses merit validation in future interventional studies. In clinical settings, PIV may inform treatment selection—patients with markedly high PIV could benefit from IL-6 or TNF blockers when standard therapies fail (37).

Subgroup analyses provided additional insight into these associations. The inverse relationship between PIV and bone turnover markers was especially pronounced among patients with hypertension, aligning with previous research linking inflammation and endothelial dysfunction in hypertension to bone loss (24). In contrast, this association was weaker in individuals with impaired renal function (elevated UN or Cr), likely reflecting confounding from renal osteodystrophy and altered BTM metabolism (23). These findings suggest PIV may be more reliable in patients with preserved kidney function. Fracture type may also affect the inflammation–bone turnover relationship: hip fractures often reflect acute inflammation, while vertebral fractures may represent chronic low-turnover states. Stratified analysis by fracture site may enhance the clinical utility of PIV. Although the P1NP association was weaker in men, possibly due to a smaller sample or sex differences, the consistent inverse *β*-CTX association across sexes supports a shared inflammatory pathway for impaired resorption (38). Weaker P1NP effects in men may relate to immune senescence, hormonal regulation, or reduced anabolic response (39).

Our findings add to growing evidence for using blood-based indices such as PIV to assess complex pathophysiology. Compared to simpler markers such as NLR or PLR, PIV offers broader prognostic value across diseases such as cardiovascular conditions, cancer, frailty, and autoimmune disorders (13, 40, 41). Related indices such as the pan-immune-inflammation index (PII) have also been associated with disease activity in rheumatoid arthritis (40) and with clinical outcomes in vasculitis (41). Extending PIV’s utility to osteoporosis suggests it captures systemic inflammation relevant to skeletal health. PIV also correlates with CRP and hypoalbuminemia—features of the CRP/albumin ratio—previously linked to increased osteoporosis risk (29).

Building on these findings, patients with osteoporotic fractures and elevated PIV may benefit from a multidisciplinary strategy combining standard osteoporosis management with anti-inflammatory interventions. Potential interventions include dietary modifications (13), adequate intake of vitamin D and antioxidants (5), and proactive management of chronic infections. While certain anti-resorptive agents may exert anti-inflammatory effects (12), it remains unclear whether baseline PIV levels can predict treatment response. Anabolic therapies such as teriparatide may be less effective in high-PIV patients, as chronic inflammation disrupts key anabolic pathways (4, 42). Nevertheless, identifying patients with a low-turnover, inflammation-driven phenotype using PIV may help inform decisions about the duration and customization of anabolic treatment plans. Compared to bone-specific markers such as osteocalcin or the OPG/RANKL ratio, PIV captures broader immune-inflammatory activity, potentially better reflecting inflammation-related skeletal fragility (43).

Several limitations should be acknowledged. First, as a cross-sectional study, causality cannot be established—elevated PIV may both contribute to and result from reduced bone turnover, or reflect shared factors such as frailty (22). However, the association persisted after adjusting for frailty indicators (e.g., albumin and BMI), suggesting a likely biological link. Second, as a single-center study conducted in a Chinese population, the applicability of these findings to other populations may be limited. However, our results are consistent with other cohorts (16, 27, 44). Third, although PIV was measured within 72 h of admission to reduce acute-phase confounding, fracture-related inflammation may still influence results. Notably, the consistent inverse association between PIV and BTMs despite this potential confounder suggests that chronic inflammation plays a more dominant role. Longitudinal studies are needed to clarify causality and assess temporal dynamics. Fourth, we did not measure cytokine levels or bone-related gene expression to directly validate the underlying mechanistic pathways. Previous studies have shown elevated levels of TNF-producing T cells and IL-17 in women with osteoporosis, both of which are associated with increased bone loss (32, 45). Although IL-17 data were unavailable, our findings support T-cell-mediated inflammation in turnover suppression. Future studies combining PIV with cytokine and transcriptomic profiling may define specific inflammatory signatures in osteoporosis. Finally, only *β*-CTX and P1NP were assessed. Other markers such as osteocalcin, bone ALP, or RANKL/OPG might provide further insight (5, 46). While we adjusted for calcium, renal, and liver function, unmeasured confounders such as PTH and 25(OH) D could affect BTMs. However, given the exclusion of secondary hyperparathyroidism and common vitamin D use in our region, their impact is likely limited.

Despite limitations, this study has notable strengths. The large sample size enhances statistical power, enabling the analysis of non-linear trends. Rigorous methods (GEEs, GAMs, threshold modeling) and extensive confounder adjustment support the robustness of results. Consistency with prior cohorts (16, 18, 24) further enhances validity. By examining both β-CTX and P1NP, we captured the suppression of both resorption and formation, offering a more integrated perspective than single-marker or BMD-only evaluations. Together, our findings suggest that osteoporotic fracture patients with elevated systemic inflammation—reflected in high PIV—represent a low-turnover subgroup. These individuals may benefit from combined anti-inflammatory and anabolic approaches. Since tools such as Fracture Risk Assessment Tool (FRAX) do not account for systemic inflammation, incorporating markers such as PIV could improve risk stratification and personalized treatment in osteoporosis (38, 47).

In conclusion, this study demonstrated a strong inverse association between PIV and bone turnover markers in patients with osteoporotic fractures, highlighting the inhibitory role of chronic inflammation in bone remodeling. These results support the potential of PIV as a systemic inflammation biomarker for osteoporosis, consistent with its role in other chronic diseases (13, 14, 40). Collectively, our findings suggest that PIV could help define an “inflammatory phenotype” among osteoporotic patients—individuals who may experience higher fracture risk and distinct treatment responses despite comparable BMD. Recognizing this subgroup could support more personalized treatment strategies, such as incorporating anti-inflammatory or immunomodulatory interventions in addition to conventional osteoporosis therapy. Future longitudinal and interventional studies are warranted to validate whether PIV can serve as a reliable biomarker for patient stratification, predict therapeutic efficacy, and monitor response to emerging immunomodulatory treatments in osteoporosis. This perspective also aligns with the concept of “immunoporosis,” which refers to osteoporosis driven by immune dysregulation, emphasizing the central role of immune–bone interactions in skeletal fragility (4).

## 5 Conclusion

In patients with osteoporotic fractures, elevated PIV is significantly associated with reduced bone formation and resorption markers, suggesting a state of suppressed bone turnover. These findings point to a potential “inflammaging” phenotype of low-turnover osteoporosis, where chronic systemic inflammation contributes to skeletal fragility. As an easily obtainable biomarker from routine blood tests, PIV may aid in identifying individuals at higher risk who could benefit from adjunctive anti-inflammatory interventions alongside conventional osteoporosis treatment. Incorporating PIV into existing fracture risk assessment tools may also enhance predictive accuracy. Overall, PIV offers a promising link between immune function and bone health, reinforcing the importance of osteoimmunology in advancing precision medicine.

## Data Availability

The original contributions presented in the study are included in the article/Supplementary material, further inquiries can be directed to the corresponding authors.
